# The Stanford Brainstorm Social Media Safety Plan (SMS): Introducing a New Tool

**DOI:** 10.2196/72057

**Published:** 2025-12-29

**Authors:** Darja Djordjevic, Nina Vasan

**Affiliations:** 1 Department of Psychiatry and Behavioral Sciences, Stanford University School of Medicine, 401 Quarry Road, Stanford, CA, 94305-5717, United States

**Keywords:** social media safety, youth, habits, tools, families, prevention

## Abstract

We propose the Stanford Brainstorm Social Media Safety Plan (SMS) as a user-friendly, collaborative, and effective tool to mitigate the imminent dangers and risks to mental health that are associated with social media use by children, adolescents, and young adults. This tool is informed and inspired by suicide safety plans as part of suicide safety planning, which have long shaped the standard of care for psychiatric discharges from inpatient units, emergency rooms, and comprehensive psychiatric emergency programs, as well as longitudinal outpatient care following occurrences of suicidal ideation or suicide attempts. In many systems including those of the Veterans Health Administration, they constitute an absolute requirement prior to the discharge of the patient. This social media safety plan is to be used proactively, in times of normalcy as well as crisis. While there are parental controls for digital devices and online platforms, official legal age requirements for online accounts, and individual parenting approaches, there is a dearth of practical tools that youth, families, schools, and communities can use to shape and alter social media use parameters, rules, and habits. Furthermore, providers in psychiatry, child and adolescent psychiatry, and mental health at large are often confronted with behaviors and issues related to social media use during time- and resource-limited appointments, providing a massive opportunity for interventions that are harm reduction–oriented and easy to disseminate. While it has not been studied in a clinical trial, we have used it extensively with patients and families, and presented it to larger audiences at mental health and technology conferences over the past two years. The responses and feedback we have received, as well as reported anecdotal experiences with using it, have been overwhelmingly positive. An already unfolding child and adolescent mental health epidemic in the United States has been observed and deepened partly by way of easy access to social media (and digital-screen time) with inadequate safeguards and monitoring in place. Social media’s impacts and related interventions require a multitiered biopsychosocial and cultural approach: at the level of the individual child, the family, the school, the state, the market, and the nation. At the level of youth and their parents or caregivers, practical tools are desperately needed. We propose the SMS as one such significant tool.

## Introduction: Social Media and American Youth

The impact of social media on youth mental health continues to be at the forefront of conversations about a national youth mental health crisis. While social media has positive features and applications, its regular use also presents significant psychological and psychiatric risks. The 4.9 billion people who use social media are in desperate need of tools that can help them change or modify their behaviors and habits around this potentially addictive technology. The Stanford Brainstorm Social Media Safety Plan (SMS) is a new tool that youth and their families can use collaboratively to address social media use, foster open dialogue about it, and build healthy habits. Suicide safety planning (SSP), a preventative tool used by clinicians to help patients manage suicidality, informed the creation of the SMS.

What is the relationship between social media use and mental health? The answer lies in consideration of *what* and *how much* one consumes. Dubbed “The Goldilocks Hypothesis,” one research study concluded that moderate digital-screen time may be beneficial for adolescent mental well-being [1,2]. It suggested that optimal use may be at the rate of one to two hours daily. Thereafter, it is likely that well-being decreases continuously as daily digital-screen time increases. Parents and caregivers struggle with setting firm limits on social media use for their children; they are often ill at ease with healthy discipline and their own efficacy in this area.

In May 2023, the Surgeon General’s Advisory on Social Media and Youth Mental Health called for multilateral efforts to address the “harm [of social media] to the mental health and well-being of children and adolescents” [3]. In June 2024, he proposed warning labels to be featured on social media platforms in order to inform parents and families in advance about the risks of social media use for adolescent mental health [4]. That same month, Governor Kathy Hochul of New York signed legislation to combat addictive social media feeds and protect children online. Named the Stop Addictive Feeds Exploitation (SAFE) For Kids Act, the law requires social media companies to restrict addictive feeds on their platforms for users under the age of 18 years [5]. As of September 2025, a school day cell phone ban has been implemented in all New York public schools [6].

In 2025, various states have enacted laws that regulate children and teens’ access to social media—these include Arkansas, Louisiana, Nebraska, Oregon, and Virginia [7]. Major federal bills aimed at bolstering children’s online safety and privacy include the Children and Teens’ Online Privacy Protection Act (COPPA 2.0) and the Kids Online Safety Act (KOSA), both of which have been under consideration in the Senate and House of Representatives for years. State-based and federal legislation constitute one important response to curtailing the potentially detrimental effects of social media use by youth.

Notwithstanding the possible harms of social media, it has positive aspects, which can be leveraged in public health efforts directed towards youth mental health. Youth who are members of minority and marginalized groups, such as those who identify as LGBTQIA+ (lesbian, gay, bisexual, transgender, queer/questioning, intersex, and asexual, with the "+" [plus] signifying all other identities [like pansexual, two-spirit, nonbinary]), have found social media provides safe spaces to explore identity and belonging [8,9]. Recent research has also explored how digital media and especially social media can be leveraged to prevent negative mental health outcomes and advance youth mental health [10].

## The Pros and Cons of Social Media Use

Worldwide, the average user spends 2 hours and 21 minutes [11] on social media each day, while the average teenager spends 5 hours and 49 minutes [12]. For the average African American or Latinx teen, add 2 hours to that. There has been mounting concern that we need more rigorous research to establish causality between social media use and adverse health risk behaviors as well as psychiatric illness in youth, while correlations have been established [13-15]. One conclusion is that social media use is correlated with alarming increases in depression, anxiety, eating disorders, self-harm, suicidal behaviors, and cyberbullying as well as biopsychosocial alterations in adolescent development [16].

Platforms feature posts wherein young people incorrectly diagnose themselves with a range of complex disorders, from autism to bipolar disorder. Social media can lead to constant comparison of oneself to others, which, for some users, results in lower self-esteem and feelings of inadequacy. Some researchers have tried to identify predictors of problematic social media use and determined that they include younger age, cyberbullying, intensity of electronic media communication, and preference for online social interaction [17].

Conversely, social media can be beneficial when used in moderation. Some studies have highlighted specific positive uses such as social media’s power for health promotion campaigns among adolescents, including those for healthy eating, exercise, and care of chronic medical conditions such as diabetes [18,19]. Others have outlined how digital media can advance youth mental health, namely by strengthening social connection, identifying risk for mental health problems, as well as disseminating mental health education and resources [8-10].

Social media can be used to disseminate information and track case rates during a pandemic, just as it can spread misinformation [20]. Scientists have articulated a call to action for more research on social media, while also asking how it can facilitate access to lifestyle coaching, peer support groups, verified mental health education tools, and the like [8-10,21-24]. It can increase human connection and build community across gulfs in geography and time—perhaps even help build tolerance [21].

## From Suicide Safety Plans to Social Media Safety

The proposed SMS is a user-friendly, collaborative, and effective tool to mitigate the imminent dangers and risks to mental health that are associated with social media use by children, adolescents, and young adults. It is informed by SSP, part of the psychiatric standard of care in managing suicidality across practice contexts. Just as SSP helps patients to conceptualize protective behavioral responses to suicidal ideation, the SMS enables the user to analyze their behaviors, and take a stepwise approach to behavioral change and reduction of risks associated with social media use. More broadly, safety plans have been adapted to address behavioral change with respect to the use of alcohol and substances as well as gambling and pornography. Patients report benefit from planning ahead, and such action-oriented plans empower them with an awareness of what to do, who to contact, and how to think when they are in a difficult or risk-laden moment.

A systematic review addressing the effectiveness of SSP found that the evidence supports improvements in suicidality, suicide-related outcomes, and treatment outcomes [25]. Another systematic review concluded that SSP is effective for reducing suicidal behavior and ideation [26]. One study of adolescents after psychiatric hospitalization determined that high-risk adolescents retain and use their safety plans [27], while another suggested that adolescents understood, liked, and believed they would use a self-guided safety plan [28]. A systematic scoping review regarding the use of SSP for children and young people found that there is promising evidence of effectiveness for safety planning for children and young people [29].

The Veterans Health Administration first introduced the use of SSP in 2008 [30]. Numerous studies demonstrate that SSP is effective for reducing suicidal behavior and ideation [25,26,31]. Other research has confirmed the overall benefits of SSP, including its importance as a tool aiding the development of mental health and primary care infrastructures, involving veterans, their peers, family members, and communities across rural and other resource-poor settings [32-38].

When patients are experiencing suicidal ideation, they have difficulty thinking clearly and even logically. Similarly, social media is a powerful force that can overwhelm the young user’s mind—trends like doomscrolling demonstrate how potent it is, making it hard for the vulnerable user to think productively and to interrupt their engagement with social media. The exercise of crafting a targeted plan for addressing behaviors empowers users to feel more in control of their use. Furthermore, just as suicidal ideation can come and go, challenges with social media use can arise intermittently and unexpectedly. Just as providers revisit SSP regularly with patients at risk, safety planning around social media use should be a regular exercise.

The SMS can be easily introduced in the classroom, clinic appointment, or youth club meeting, and then further pursued when time allows rather than needing to be completed immediately in one sitting. One study showed that there was greater self-knowledge of warning signs over time among participants who were given access to SSP with a mobile app component [39], while another found that mobile apps were preferred in times of crisis but perhaps not in the long term [28]. The SMS could easily be adapted for mobile and digital apps.

## The Stanford Brainstorm Social Media Safety Plan

**Figure 1.**
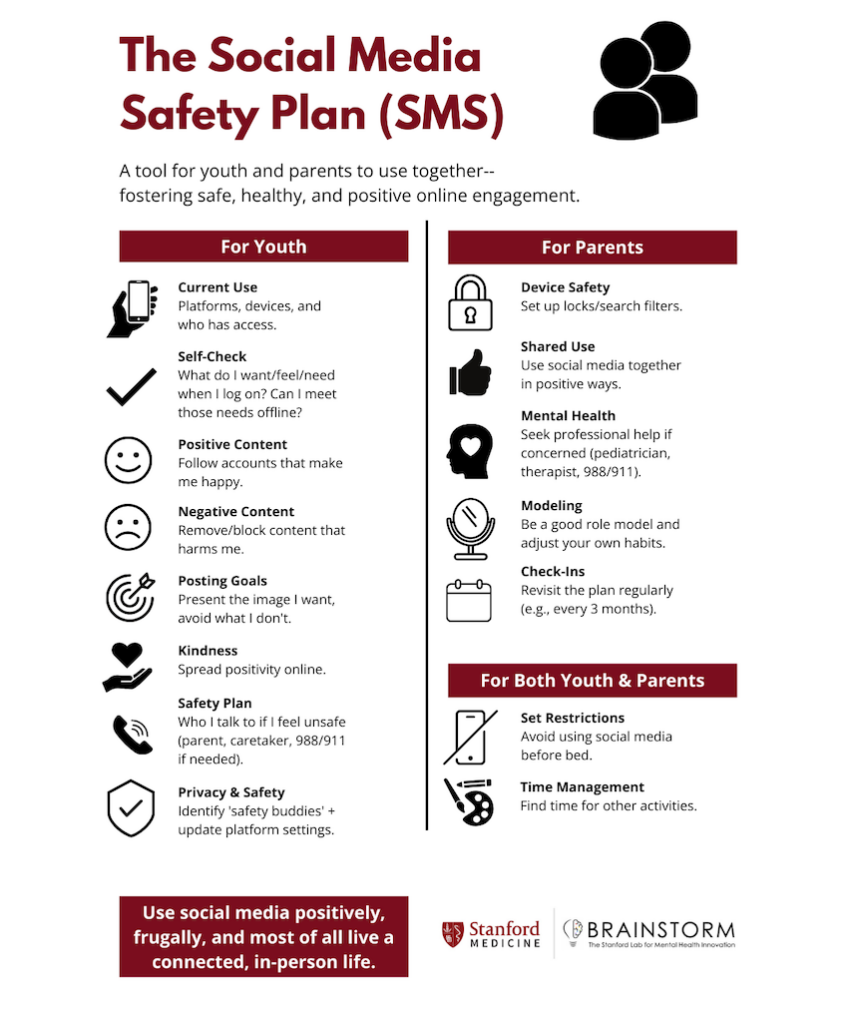
The Stanford Brainstorm Social Media Safety Plan.

Extrapolating from SSP practices, the proposed recommendation is that every social media user who has experienced harm, fear, or compromised safety create a SMS. It should also serve as a preventative measure and exercise for social media users who have not yet experienced any negative effects of their use (Figure 1).

The SMS is to be used proactively, in times of normalcy as well as crisis. While parental controls for digital devices and online platforms, official legal age requirements for online accounts, and individual parenting approaches abound, there is a dearth of practical tools that youth, families, schools, and communities can use to shape and alter social media use parameters, rules, and habits. Furthermore, mental health providers are often confronted with behaviors and issues related to social media use during time- and resource-limited appointments, providing a massive opportunity for interventions that are harm reduction–oriented and easy to disseminate. Proposed herein is one such tool, the SMS. While it has not yet been studied in a clinical trial, it has been used by patients and families for the past 2 years. When presented to larger audiences at mental health and technology conferences, the responses and feedback received, as well as anecdotal evidence collected, have been overwhelmingly positive.

The SMS was originally conceived for youth, yet adults have also found it useful. The SMS draws upon the most prevalent issues and challenges seen in daily clinical practice. It has been used in a broad variety of practice contexts, from inpatient units and emergency rooms to clinics and school-based mental health programs, and with patients in urban and suburban communities.

The plan was conceived to maximize open conversation and collaboration between parents or caregivers and children. It allows users to identify their triggers as well as contemplate ways to engage with positive content online and eschew negative content. Its questions are also meant to inspire discussion around online kindness and digital citizenship, and even shared engagement online wherein kids and parents consume content side-by-side. Parents and caregivers are encouraged to use search engine or phone locks. One question in the plan encourages the conversion of online interests into in-person hobbies; another question galvanizes kids and families to identify the need for social media and digital screen-time weans or vacation periods (Multimedia Appendix 1).

A child and adolescent mental health epidemic in the United States has been increasing partly by way of easy access to social media (and digital-screen time) with inadequate safeguards and monitoring in place. Legal regulation of tech companies and their social media platforms remains controversial, yet the authors are in support of it. Social media’s impacts and related interventions require a multitiered biopsychosocial and cultural approach: at the level of the individual child, the family, the school, the state, the market, and the nation. At the level of youth and their parents or caregivers, practical tools are desperately needed. The SMS is one such significant tool. The authors welcome any feedback from those who use it.

## Supplementary material

10.2196/72057 Multimedia Appendix 1 The Stanford Brainstorm Social Media Safety Plan.

## Abbreviations

COPPA: Children and Teens’ Online Privacy Protection Act
KOSA: Kids Online Safety Act
LGBTQIA+: lesbian, gay, bisexual, transgender, queer/questioning, intersex, and asexual, with the "+" [plus] signifying all other identities [like pansexual, two-spirit, nonbinary)
SAFE: Stop Addictive Feeds Exploitation
SMS: Stanford Brainstorm Social Media Safety Plan
SSP: suicide safety planning
