# Supplementary material for: The Stanford Brainstorm Social Media Safety Plan (SMS): Introducing a New Tool
Source: JMIR Ment Health. 2025 Dec 29;12:e72057. doi: 10.2196/72057 (PMC12747419; doi:10.2196/72057)
Supplement: Multimedia Appendix 1 [file mental-v12-e72057-s001.docx]

**APPENDIX:** **The Stanford Brainstorm Social Media Safety Plan (SMS)**

Directions: The first important aspect of creating a social media safety plan is that it be a conversation with youth and trusted adult(s), which could include parents, guardians, close relatives/caretakers, older siblings, teachers, coaches, counselors, etc. Talk to your children about your own social media use and how it impacts you: this is important to normalize negative feelings and the need for healthy behaviors, applicable to adults too. Set aside 30-60 minutes to talk to children for an initial conversation, and then follow up every three months, and whenever issues come up for your child, their friends, or in the news. Like spring cleaning, this is best as a regular activity to clean out your feeds and accounts.

This is an especially valuable exercise that can be completed in school with the participation of an entire class, and could even involve inviting a social media expert in to address students.

If your child is resistant to this conversation, help them understand that this is about their health and safety, and furthermore, as a parent, it’s your responsibility to help and protect them with respect to social media use, like many other areas of life. This conversation should not be connected to punishment. Instead, it should occur during a peaceful period of time, as the goal is for youth to open up and be in a good headspace when creating the safety plan. This conversation should be appropriate to the child’s age, developmental stage, communication style, and personality. As we say in psychiatry, meet them where they are.

One of the main goals here is for youth to develop an awareness of how social media makes them *feel* *and what they end up doing as a result of those feelings* (ex: “When I see pictures of celebrities at the beach, it makes me feel insecure about my body. I then tell myself that I’m not good enough or beautiful or fit enough, and then I go to my room, close the door, and skip family dinner.”)

Lastly, while we initially created this for youth given the urgent need, this is also valuable for adults - meaning all users—as we’re all susceptible to the risks and negative effects of social media. By answering the questions below, you and your child can start to create your personalized Stanford Brainstorm Social Media Safety Plan (SMS).

**The Stanford Brainstorm Social Media Safety Plan**

**From the youth’s perspective (encouraging self-reflection and validating their evolving thoughts, emotions, feelings, ideas, and dreams):**

1. I currently use these social media platforms: _________________. I access them on the following devices: ___________________. In addition to myself, ___________(no one vs. name the parent or guardian who is supervising/also has access) has access to my devices.

1. When I sign on to social media platforms, what am I wanting? What do I need? What am I feeling? What am I thinking about? How can I get these needs met *without* social media?

1. The content on social media that makes me happy, laugh, or generally improves my mood: ________________. I will aim to follow more accounts featuring this content.

1. The content on social media that makes me feel depressed, anxious, stressed, triggered, or lowers my self-esteem:  ______________. I will remove or block these accounts.

1. When I post my own content online, I want to present an image of __________________ and avoid ____________.

1. I can be kind and spread positivity online by ________________.

1. If I feel unsafe about a person I meet online or content I consume, I will talk to ______________. I will also immediately alert ____________ [any parent or caretaker who has access to child’s devices, as named in prompt #1]. If I feel unsafe and my parent(s), teacher(s), or other caretakers are not physically present, I will send them a text message or screenshot. If I am feeling overwhelmed by my own thoughts, emotions, or feel at risk of self-harm or harm by someone else, I will call 988, the national and local mental health crisis hotline, or 911 if I need immediate in-person aid.

1. I will protect my personal privacy and general safety by naming the following safety buddies in addition to my parent(s): ________. I will call, text, or seek them out in person during an acute situation that makes me feel unsafe (as in prompt 8). I will read how each platform uses my information, and change the settings in a way that meets my safety goals and those of my parents or guardians. These may include limiting access to my profile or minimizing the amount of identifiable or personal information or content on my profile as follows: __________.

1. I will limit my social media use to these platforms: ________. I will stop using these platforms: _________.

1. [As necessary.] I will collaborate with my parent/guardian to wean myself off of the following social media platform(s) with the following wean schedule: ___________.

1. [As necessary.] I will take a full social media vacation during the following period of time: [list dates] _____________.

1. I will avoid spending more than _____ hours per day on social media. I will stop using social media by ____ PM on weekdays and by _____ PM on weekends. When I realize I am scrolling mindlessly, endlessly, or feel addicted to social media, I will take the following actions to stand up and help myself switch to the following different activities: ______________.

1. I will place my phone in the following location 1-2 hours before bedtime: ______ I pledge to not go to bed with my phone. I will aim to be in bed by ____ and get ___ hours of sleep each night. (We recommend ending social media time at least one hour before bedtime to avoid sleep hygiene disruptions and help kids learn other bedtime-appropriate routines including personal hygiene and dental care, putting on PJs, dimming lights, engaging with a book or soothing music, or in-person talk and storytelling.)

1. If I spend less time on social media, I’ll have more time for _________, which is something I care about and which is important to my sense of self, and/or overall well-being.

1. If I see something on a social media platform that could become a screen-free activity, hobby, or interest to explore, I will bring it up with my parent(s) and family members (including siblings). There are countless examples: sports and outdoor activities, board or card games, music, dance, paints, outdoor adventures, books, magazines or series including anime or manga, new foods, fashion, collectible items, contests that involve a new project or hobby, a bicycle, or anything that gets you curious or builds your dreams!

**From the parent’s perspective:**

1. I will use the following search engine or phone locks on my child’s devices: [list each device and the corresponding locks] _______________________________________.

1. My child and I will use social media together in the following positive ways, so that we sometimes consume and discuss the same content in real-time, or use it to bolster an educational activity, hobby, interest, or issue-based discussion: ____________________________________________________________________.

1. If I’m worried about my or my child’s mental health, well-being, or safety, I will contact [medical professionals such as the child’s pediatrician, and therapist/mental health professional where applicable] and access professional help for them ASAP. I will also make use of 988 and 911 as necessary, and as described in prompt 8.

1. I will endeavor to serve as a good role model for my child in my own use of social media, technology, and personal digital devices. To this end, I will make the following changes to my habits: _____________. I will continue the following positive habits I cultivate in this area of daily life: _____________.

1. I will revisit this safety plan with my child, reaffirm it or revise it however necessary, at regular intervals. For now, I will do so every _____ weeks.

Additional prompts can be added as desired: Create your own statements based on the specific issues that arise for you. For example, if you struggle with body image, you may seriously consider a social media vacation from all posts or accounts followed that feature beauty, fashion, and body shape/weight-focused content. If you struggle with an over-dependence or addiction to gaming, pornography, or gambling-inclusive platforms, you might consider a short vacation from them under the supervision of the parent, but with a clear plan for some positive and healthful reward to replace that time such as an IRL activity with a friend or parent.

To summarize these recommendations, when it comes to social media: Use social media positively, frugally, and most of all live a connected, in-person life. Youth, parents and loved ones should be actively involved in developing and implementing a social media safety plan. An essential ‘SMS’ for the future!
